# The effect of neprilysin and renin inhibition on the renal dysfunction following ischemia‐reperfusion injury in the rat

**DOI:** 10.14814/phy2.14723

**Published:** 2021-03-14

**Authors:** Fayez T. Hammad, Suhail Al‐Salam, Sarah S. AlZaabi, Maryam M. Alfalasi, Awwab F. Hammad, Javed Yasin, Loay Lubbad

**Affiliations:** ^1^ Department of Surgery United Arab Emirates University Al Ain United Arab Emirates; ^2^ Department of Pathology United Arab Emirates University Al Ain United Arab Emirates; ^3^ College of Medicine & Health Sciences United Arab Emirates University Al Ain UAE; ^4^ School of Medicine University of Jordan Amman Jordan; ^5^ Department of Internal Medicine College of Medicine & Health Sciences United Arab Emirates University Al Ain United Arab Emirates

**Keywords:** ischemia‐reperfusion injury, neprilysin inhibition, renal functions, renin inhibition

## Abstract

The natriuretic peptide (NP) system counter‐regulates the renin‐angiotensin system (RAS), so enhancing the activity of natriuretic peptides (NPs) may be beneficial in conditions when RAS is activated such as ischemia‐reperfusion injury (IRI). Neprilysin is the key enzyme responsible for the degradation of NPs. The effects of neprilysin inhibition or the combination of neprilysin inhibition and RAS inhibition on renal IRI‐induced renal dysfunction have not been investigated yet. To investigate this, rats underwent sham surgery or bilateral IRI for 20 min. G‐Als, G‐Scb, and G‐Als+Scb underwent similar protocol but received aliskiren (renin inhibitor), sacubitril (neprilysin inhibitor) or a combination of both pre‐ and post‐IRI, respectively. IRI caused significant alterations in all renal functional parameters, markers of acute renal injury, pro‐inflammatory and pro‐fibrotic cytokines, and histological features. All these alterations were significantly attenuated in G‐Als, G‐Scb, and G‐Als+Scb. The attenuations in the alterations in serum creatinine, creatinine clearance, and histological features were larger in G‐Als+Scb compared to either G‐Als or G‐Scb. We conclude that RAS blockade by a renin inhibitor (aliskiren) or neprilysin inhibition by sacubitril separately led to significant attenuation in the renal IRI‐induced renal dysfunction. The combination of aliskiren and sacubitril was more effective than either one alone.

## Graphical abstract

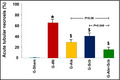

## 1 INTRODUCTION

Renal ischemia‐reperfusion injury (IRI) is an invariable consequence of several conditions including renal transplantation and resuscitation following systemic hypotension (Weight et al., 1996). Efforts to decrease the impact of IRI have been the subject of many previously performed studies and several agents and technique's modifications were shown to attenuate the IRI‐induced renal injury (Goes et al., 1995; Hammad, Davis, et al., 2001a, Hammad, Wheatley, et al., 2001b; Petersen & Mitchell, 1981; Troppmann et al., 1995; Unal et al., 2001; Yang et al., 2001).

The renin–angiotensin system (RAS) plays an important role in the IRI‐induced renal alterations. Inhibition of the activity of RAS was shown to have renal protective effects in IRI (Bhalodia et al., 2010; Efrati et al., 2011; Fouad et al., 2010; Habibey et al., 2008; Kontogiannis & Burns, 1998; Molinas et al., 2009). RAS can be blocked at different steps including inhibition of angiotensin‐converting enzyme and blockade of angiotensin receptors (Bhalodia et al., 2010; Cagnoni et al., 2010; Efrati et al., 2011; Fouad et al., 2010; Habibey et al., 2008; Kontogiannis & Burns, 1998; Molinas et al., 2009). Inhibition of the RAS can also be achieved by inhibiting renin, the rate‐limiting step in the formation of angiotensin‐I and therefore reducing the formation of all downstream products of the RAS system (Cagnoni et al., 2010; Feldman, 2010). Aliskiren, the first oral direct renin inhibitor which has been approved for the treatment of hypertension by the US Food and Drug Administration (Morganti & Lonati, 2011), was shown to attenuate the IRI‐induced renal dysfunction (Hammad et al., 2013).

The natriuretic peptide (NP) system is a neurohormonal system that counter‐regulates the RAS. Therefore, enhancing the activity of natriuretic peptides (NPs) may be beneficial when RAS is activated in certain conditions. NPs are a family of three peptides that include atrial (ANP), brain (BNP), and c‐type (CNP; Judge et al., 2015; Sager, 2004). Neprilysin (also known as neutral endopeptidase (NEP) is the key enzyme responsible for the degradation of NPs (Wilkins et al., 1997). NEP is a membrane‐bound zinc‐containing metalloproteinase which is widely spread in various tissues including brain, vascular endothelial cells, and cardiac myocytes but has a great abundance in the brush border of renal proximal tubules (Benigni et al., 2004; Mangiafico et al., 2013).

In renal IRI, the administration of atrial natriuretic peptide (ANP) has been shown to cause attenuation in the IRI‐induced renal dysfunction (Chujo et al., 2008; Mitaka et al., 2014; Moriyama et al., 2016). ANP is not available in the oral form which is more convenient to administer, at least in the clinical practice. Sacubitril is an oral neprilysin inhibitor, which results in the potentiation of the plasma ANP activity (Gu et al., 2010). The effect of neprilysin inhibitors such as sacubitril on the renal alterations following IRI has not been previously investigated.

A limited number of studies have investigated the combined effect of RAS inhibition and neprilysin inhibition in the treatment of some cardiovascular and renal diseases and this combination was shown to be promising because it might result in a better treatment outcome without necessarily increasing the side effects (Damman et al., 2018; Habibi et al., 2019; Hubers & Brown, 2016; Packer et al., 2018). In these studies, neprilysin inhibitors were used in combination with either angiotensin‐converting enzyme inhibitors or angiotensin receptor blockers. However, the use of renin inhibition in combination with neprilysin inhibition has never been investigated in any renal condition. Therefore, this study aimed at investigating the effect of neprilysin inhibition by sacubitril and the effect of the combination of renin inhibition by aliskiren and neprilysin inhibition by sacubitril on the renal dysfunction in a rat model of renal IRI.

## 2 MATERIALS AND METHODS

Studies were performed in male Wistar rats weighing 241–254 g at the time of IRI. Rats were kept in a 12‐h light–dark cycle at 20°C. They were fed a standard rat chow and had free access to water. Animals were fasted for 12 h before the experimental procedures but had water ad libitum. The experimental protocol was approved by the local animal research ethics committee.

### 2.1 Ischemia‐reperfusion injury

Under aseptic conditions, animals were anesthetized with intraperitoneal injection of ketamine hydrochloride (80 mg/kg, Pantex Holland B.V.) and xylazine Hydrochloride (8 mg/kg, Troy Laboratory PTY Limited). The left and right renal arteries and veins were exposed and dissected via a midline incision using a surgical microscope.

Renal ischemia was performed by applying microsurgical non‐traumatic bulldog clamps on both renal arteries followed by the veins. After 20 min of ischemia, the venous clamp was released followed by the arterial one. Sham animals underwent dissection of the renal arteries and veins without causing any ischemia. The wound was then closed in layers.

Forty‐eight hours following IRI, the animals were euthanized with an overdose of barbiturate and the kidneys were collected and weighed for later histological examination and measurement of inflammatory markers and cytokines.

### 2.2 Experimental protocol and sacubitril, aliskiren, and vehicles administration

Aliskiren (Novartis Pharma Stein AG, Stein) was dissolved in 0.5 ml of normal saline and administered by gavage immediately after preparation as a single daily dose of 60 mg/kg.

Sacubitril (MuseChem) was suspended in 2 ml of 0.5% carboxymethylcellulose in distilled water and 30 mg/kg was administered twice daily by gavage immediately after preparation. Aliskiren was given at least 1 h after the first sacubitril dose to avoid any possible drug interactions. Sham‐operated animals received similar volumes of the vehicles at timings similar to the treated groups.

**FIGURE 1:**
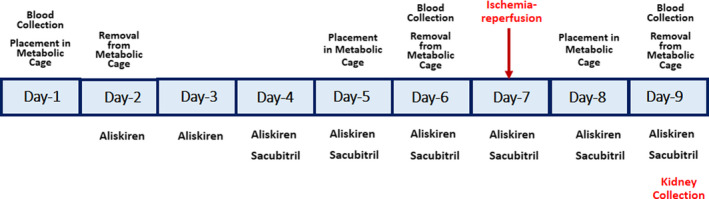
Experimental protocol showing the time points of administering the medications, blood collections, and urine collection by metabolic cages

Treatment of sacubitril and aliskiren was commenced 6 and 4 days, before the IRI, respectively. The treatment continued daily for 2 days thereafter until the sacrifice of the animals and kidney collection (Figure 1).

As shown in Figure 1, blood was collected from the tail vein to measure serum creatinine and urea at three stages: before starting any medication (basal values), just before the IRI (Pre‐IRI values), and 48 h (Post‐IRI values). Rats were placed in metabolic cages at the same stages to collect urine for measurement of urine volume and levels of albumin and creatinine.

### 2.3 Experimental groups

Animals were randomized into five groups:

1. G‐Sham (*n* = 13): Rats which underwent sham surgery and received the vehicles only.
2. G‐IRI (*n* = 13): Rats which underwent bilateral IRI and received the vehicles only.
3. G‐Als (*n* = 14): Rats which underwent bilateral IRI and received aliskiren only.
4. G‐Scb (*n* = 14): Rats which underwent bilateral IRI and received sacubitril only.
5. G‐Als+Scb (*n* = 13): Rats which underwent bilateral IRI and received both aliskiren and sacubitril.

### 2.4 Gene expression analysis

A wedge from the middle part of each kidney containing both cortex and medulla was excised, snap‐frozen in liquid nitrogen and stored at −80°C for a later measurement of gene expression of the following:

1. Markers of acute kidney injury: Kidney injury molecule‐1 (KIM1) and neutrophil gelatinase‐associated lipocalin (NGAL).
2. Pro‐inflammatory and pro‐fibrotic cytokines: Transforming growth factor‐β (TGF‐β1), plasminogen activator inhibitor‐1 (PAI‐1) and monocyte chemoattractant protein‐1 (MCP‐1)
3. The pro‐apoptotic gene p53.

Total RNA was extracted from frozen samples using Qiazol Lysis reagent (Qiagen) according to manufacturer's protocol. Quantity and quality of extracted RNA samples were estimated using NanoDrop 2000 Spectrophotometer (Thermo Fisher Scientific Inc).

First‐strand complementary DNA (cDNA) was prepared in duplicates from 1.0 µg of Extracted RNA using QuantiTect® reverse transcription kit (Qiagen) according to manufacturer protocol. The kit's protocol included genomic DNA removal using the supplied gDNA Wipeout buffer which ensured elimination of interference of genomic DNA in real‐time PCR. Prepared cDNA was used as a template for relative gene expression analysis using the Taqman® hydrolysis probe chemistry. The reaction mixture contained 75 ng cDNA, TaqMan Universal master mix (Thermo Fisher Scientific Inc), 0.5 µM of forward and reverse primers and 0.25 µM of fluorescent probe (primers and probes from Biosearch Technologies Inc.).

The probes were FAM‐labeled probes. Peptidylprolyl Isomerase A (PPIA) house‐keeping gene was used for normalization and its probe was labeled with Quasar 670 to enable multiplexing with genes of interest. All samples were run in duplicates and all prober controls were included. At least one primer of all designed PCR primers sets was spanning exon‐exon junction to further exclude any interference of genomic DNA. Sequences of primers and probes are listed in Table 1.

**TABLE 1:** Forward and reverse primers and fluorogenic probe sequences used for real time quantitative PCR analysis

KIM‐1	Forward	GCCTGGAATAATCACACTGTAAG
	Reverse	GCAACGGACATGCCAACATAG
	Probe	d FAM‐TCCCTTTGAGGAAGCCGCAGA‐BHQ‐1
Lcn2 (NM_130741.1)	Forward	CTGTTCCCACCGACCAATGC
	Reverse	CCACTGCACATCCCAGTCA
	Probe	FAM‐TGACAACTGAACAGACGGTGAGCG‐BHQ‐1
TGF‐β1	Forward	GTGGCTGAACCAAGGAGACG
	Reverse	CGTGGAGTACATTATCTTTGCTGTC
	Probe	FAM‐ACAGGGCTTTCGCTTCAGTGCTC‐BHQ‐1
PAI‐1 (NM_012620.1)	Forward	GGCACAATCCAACAGAGACAA
	Reverse	GGCTTCTCATCCCACTCTCAAG
	Probe	FAM‐CCTCTTCATGGGCCAGCTGATGG‐BHQ‐1
MCP‐1	Forward	GCTGTCTCAGCCAGATGCAG
	Reverse	CCAGCCGACTCATTGGGA
	Probe	FAM‐CCCACTCACCTGCTGCTACTCA‐BHQ‐1
p53 (NM_030989.3)	Forward	CGAGATGTTCCGAGAGCTGAATG
	Reverse	GTCTTCGGGTAGCTGGAGTG
	Probe	FAM‐CCTTGGAATTAAAGGATGCCCGTGC‐BHQ‐1

Calculated Cycle Threshold (CT) values were used to estimate changes in gene expression of target genes using delta–delta CT formula.

Results were expressed as the mean fold change in gene expression compared to the sham.

### 2.5 Histological studies

Kidneys were excised, washed with ice‐cold saline, blotted with filter paper and weighed. Each kidney was cassetted and fixed directly in 10% neutral formalin for 24 h, which was followed by dehydration in increasing concentrations of ethanol, clearing with xylene and embedding with paraffin. Three‐*μ*m sections were prepared from paraffin blocks and stained with hematoxylin and eosin. The stained sections were evaluated by the second author (histopathologist) using light microscopy in a blind fashion.

The microscopic scoring was performed by measuring the extent of necrotic area in the cortical and medullary tubules on a scale of 0–4 (0: no necrosis; 1: focal necrotic areas of less than 25%; 2: necrotic from 26% to 50%; 3: necrotic area from 51% to 75%; 4: necrotic area from 76% to 100%). Image J software (NIH) was used to measure the extent of necrosis.

### 2.6 Statistical analysis

Statistical analysis was performed using SPSS V16.0. Results were expressed as means ± SEM. One‐way factorial ANOVA and paired T‐test were used for comparison of variables between the groups and within each group, respectively. *p* value of less than 0.05 was considered statistically significant.

## 3 RESULTS

As shown in Tables 2 and 3, the basal serum creatinine, serum urea, creatinine clearance, 24‐h urinary albumin, and albumin/creatinine ratio were similar in all the groups. Similarly, there was no difference in any of these variables between the Pre‐IRI and basal values in all the groups.

**TABLE 2:** Serum creatinine, serum urea, and creatinine clearance in all the groups before the administration of the medications or the vehicle (basal values), after the administration of the medications or vehicles and just before the ischemia‐reperfusion injury (Pre‐IRI) and 2 days following IRI (Post‐IRI)

		Group				
		G‐Sham	G‐IRI	G‐Als	G‐Scb	G‐Als+Scb
S. Creatinine (mg/dl)	Basal	0.20 ± 0.02	0.21 ± 0.02	0.20 ± 0.03	0.18 ± 0.02	0.19 ± 0.02
	Pre‐IRI	0.21 ± 0.02	0.22 ± 0.03	0.18 ± 0.02	0.20 ± 0.02	0.19 ± 0.02
	Post‐IRI	0.22 ± 0.01	0.66 ± 0.10^a^	0.24 ± 0.02^a^, ^b^	0.29 ± 0.01^a^, ^b^	0.24 ± 0.02^b^
S. Urea (mg/dl)	Basal	35.1 ± 3.6	35.5 ± 3.4	34.5 ± 3.9	31.8 ± 3.2	32.4 ± 2.9
	Pre‐IRI	34.7 ± 3.4	35.0 ± 3.3	32.2 ± 2.4	32.9 ± 1.8	31.2 ± 3.0
	Post‐IRI	36.2 ± 1.7	93.8 ± 18.1^a^	44.8 ± 1.9^a^, ^b^	49.0 ± 2.4^a^, ^b^	47.3 ± 2.6^a^, ^b^
Creatinine Clearance (ml/min)	Basal	1.89 ± 0.23	1.91 ± 0.22	1.83 ± 0.26	2.00 ± 0.24	1.88 ± 0.28
	Pre‐IRI	1.76 ± 0.21	1.97 ± 0.22	1.89 ± 0.25	2.06 ± 0.22	1.82 ± 0.23
	Post‐IRI	1.99 ± 0.19	1.04 ± 0.18^a^	1.60 ± 0.16^b^	1.25 ± 0.10^a^, ^b^	1.59 ± 0.22^b^

^a^ Indicates statistical significance when compared to the Pre‐IRI within the same group.

^b^ Indicates statistical significance when compared to the G‐IRI group. Values are expressed as mean±SEM.

**TABLE 3:** Twenty‐four‐hour urinary albumin and albumin/creatinine ratio in all the groups before the administration of the medications or the vehicle (basal values), after the administration of the medications or vehicles and just before the ischemia‐reperfusion injury (Pre‐IRI) and 2 days following IRI (Post‐IRI)

		Group				
		G‐Sham	G‐IRI	G‐Als	G‐Scb	G‐Als+Scb
24‐h Urinary Albumin (µg)	Basal	0.055 ± 0.0054	0.057 ± 0.006	0.067 ± 0.005	0.066 ± 0.009	0.067 ± 0.006
	Pre‐IRI	0.062 ± 0.008	0.067 ± 0.003	0.066 ± 0.003	0.065 ± 0.005	0.069 ± 0.009
	Post‐IRI	0.058 ± 0.007	0.935 ± 0.155^a^	0.513 ± 0.068^a^, ^b^	0.703 ± 0.081^a^, ^b^	0.344 ± 0.031^a^, ^b^
Albumin/Creatinine Ratio	Basal	11.2 ± 2.7	12.4 ± 1.8	13.2 ± 1.7	11.9 ± 1.6	13.7 ± 1.2
	Pre‐IRI	11.3 ± 1.5	11.5 ± 0.91	13.7 ± 1.2	12.4 ± 1.6	14.1 ± 1.5
	Post‐IRI	11.6 ± 2.1	171.9 ± 36.3^a^	97.6 ± 17.6^a^, ^b^	138.6 ± 16.6^a^, ^b^	74.3 ± 9.0^a^, ^b^

^a^ Indicates statistical significance when compared to the Pre‐IRI within the same group.

^b^ Indicates statistical significance when compared to the G‐IRI group. Values are expressed as mean ± SEM.

In the G‐Sham, as expected, there was no difference in any of the variables post‐IRI compared to the Pre‐IRI value (*p* > 0.05 for all variables). In the G‐IRI, IRI caused a significant deterioration in renal parameters compared to the Pre‐IRI values. For example, IRI led to the rise of serum creatinine from 0.22 ± 0.03 to 0.66 ± 0.10 mg/dl (*p* = 0.04; Table 2). Creatinine clearance has dropped to 1.04 ± 0.18 compared to 1.97 ± 0.22 ml/min in the Pre‐IRI (*p* = 0.002). IRI has also led to an increase in the urinary albumin leak so that the 24‐h urinary albumin and albumin/creatinine ratio has increased to 0.935 ± 0.155 µg (vs. 0.058 ± 0.007, *p* = 0.0001) and 171.9 ± 36.3 (vs. 11.5 ± 0.91, *p* = 0.009), respectively (Table 3).

Administration of aliskiren, sacubitril, or the combination of aliskiren and sacubitril significantly attenuated the IRI‐induced alterations in these parameters as shown by comparing the Post‐IRI values in G‐Als, G‐Scb, and G‐Als+Scb groups with the Post‐IRI value in G‐IRI (Tables 2 and 3, *p* < 0.05 for all).

Despite the attenuation effect in all the three intervention groups, the combination of aliskiren and sacubitril led to the return of two parameters (serum creatinine and creatinine clearance) to values which were not different from the Pre‐IRI value (*p* > 0.05 for both). In G‐Als, out of the five parameters, only creatinine clearance returned to a value which was similar to the IRI value. Despite the significant attenuation effect of sacubitril, none of these parameters returned values similar to the Pre‐IRI value.

### 3.1 Gene expression analysis results

**FIGURE 2:**
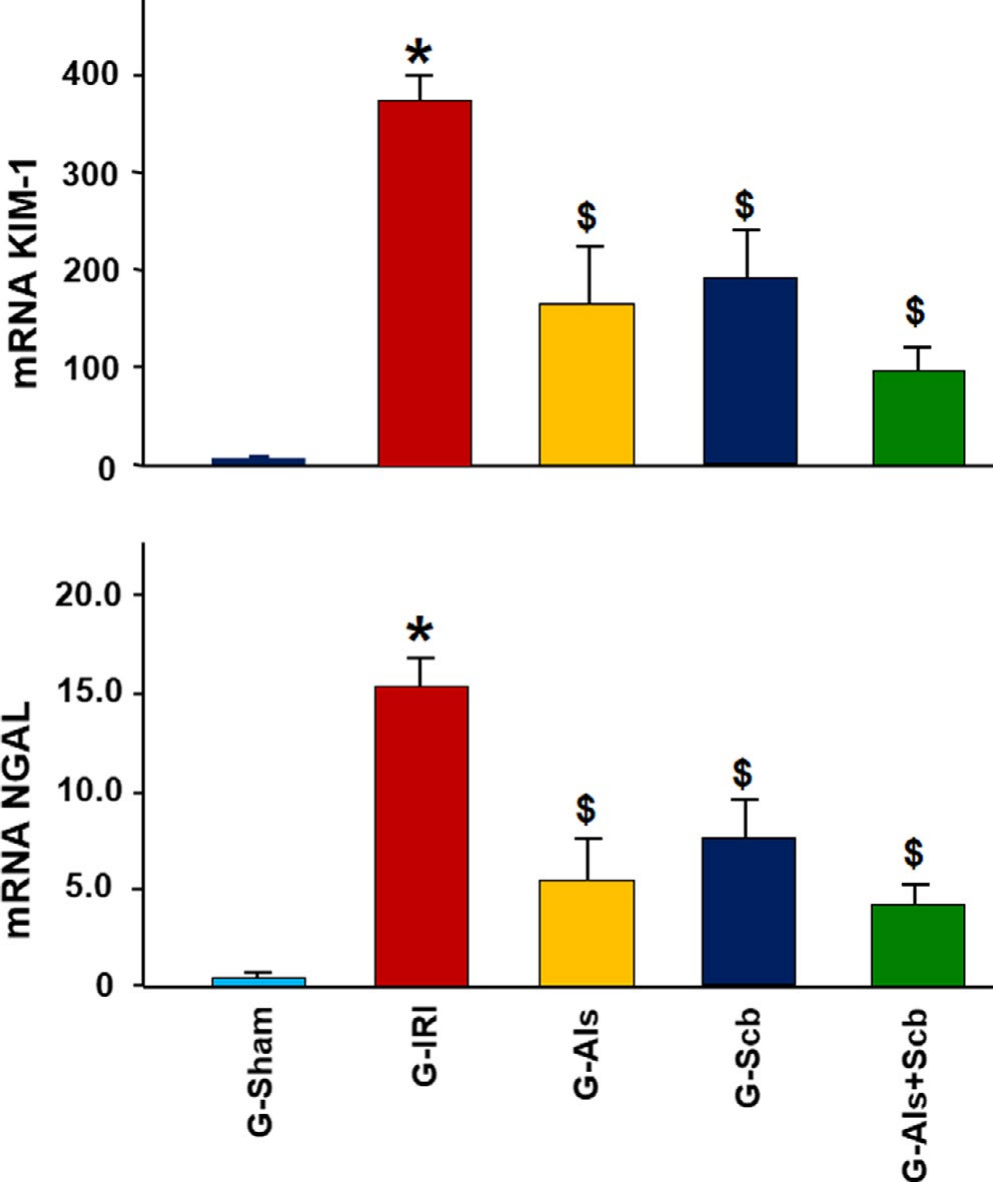
The gene expression of two of markers of acute renal injury (KIM‐1 and NGAL) in all the groups. Values represent mean ± SEM. * and $ indicate statistical significance when compared with the G‐Sham and G‐IRI, respectively

As demonstrated in Figure 2, IRI led to a significant increase in the gene expression of KIM‐1 and NGAL as shown by comparing G‐IRI to G‐Sham (374.8 ± 25.6 vs. 1.0 ± 0.3, *p* = 0.0001 and 15.4 ± 1.5 vs. 1.0 ± 0.2, *p* = 0.0004, respectively). Aliskiren caused a significant decrease in the gene expression of both markers (166.4 ± 71.4 vs. 374.8 ± 25.6, *p* = 0.03 and 5.8 ± 1.9 vs. 15.4 ± 1.5, *p* = 0.004, respectively). Similar findings were obtained in G‐Scb and G‐Als+Scb (187.5 ± 53.6 vs. 374.8 ± 25.6, *p* = 0.017 and 7.4 ± 1.6 vs. 15.4 ± 1.5, *p* = 0.001, respectively). The expression of both markers, was not significantly different when the combination therapy was compared to each agent alone (*p* > 0.05 for all comparisons).

**FIGURE 3:**
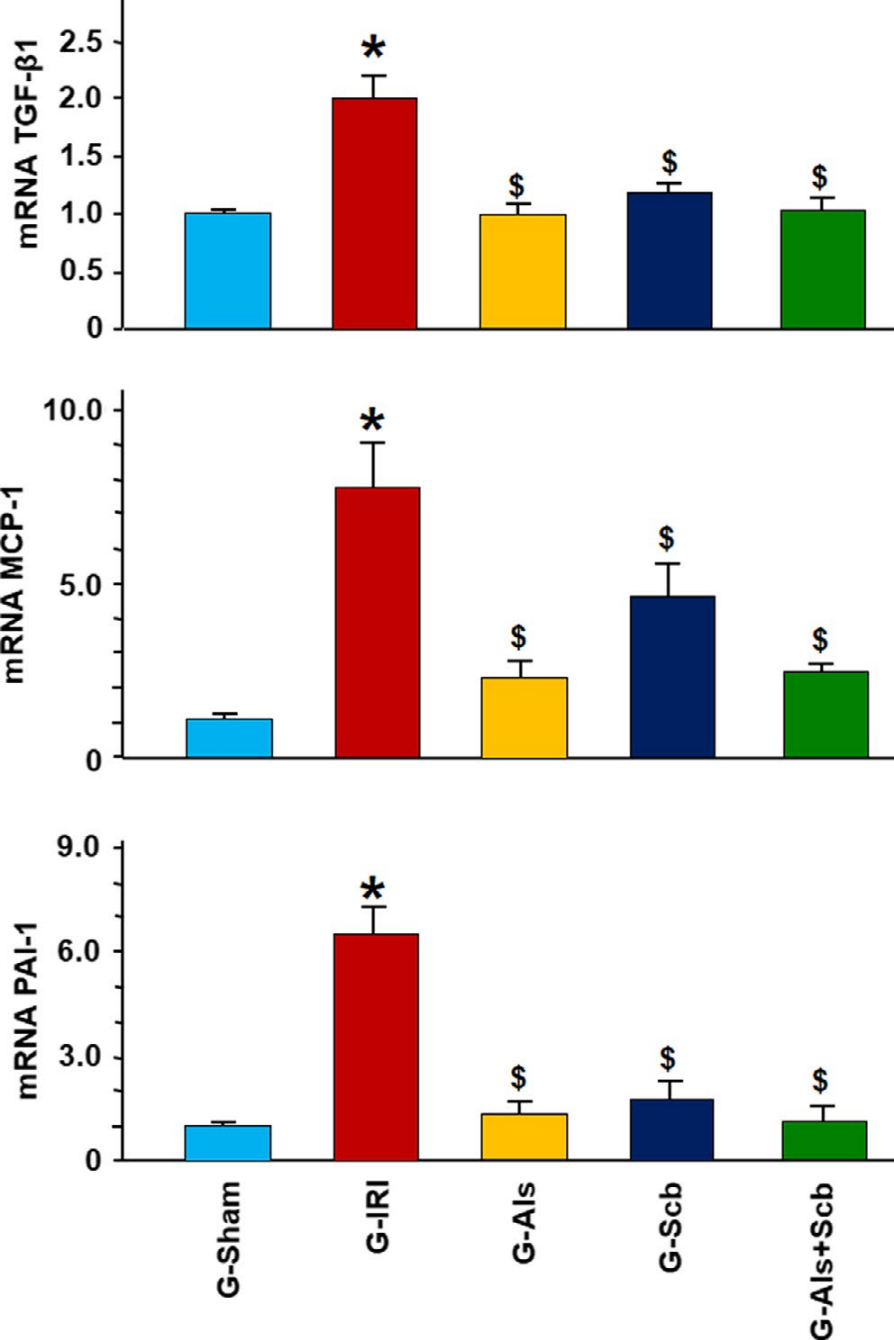
The gene expression of the pro‐inflammatory and pro‐fibrotic cytokines TGF‐β1, PAI‐1, and MCP‐1 genes in all groups. Values represent mean ± SEM. _*_ and $ indicate statistical significance when compared to the G‐Sham and G‐IRI, respectively

**FIGURE 4:**
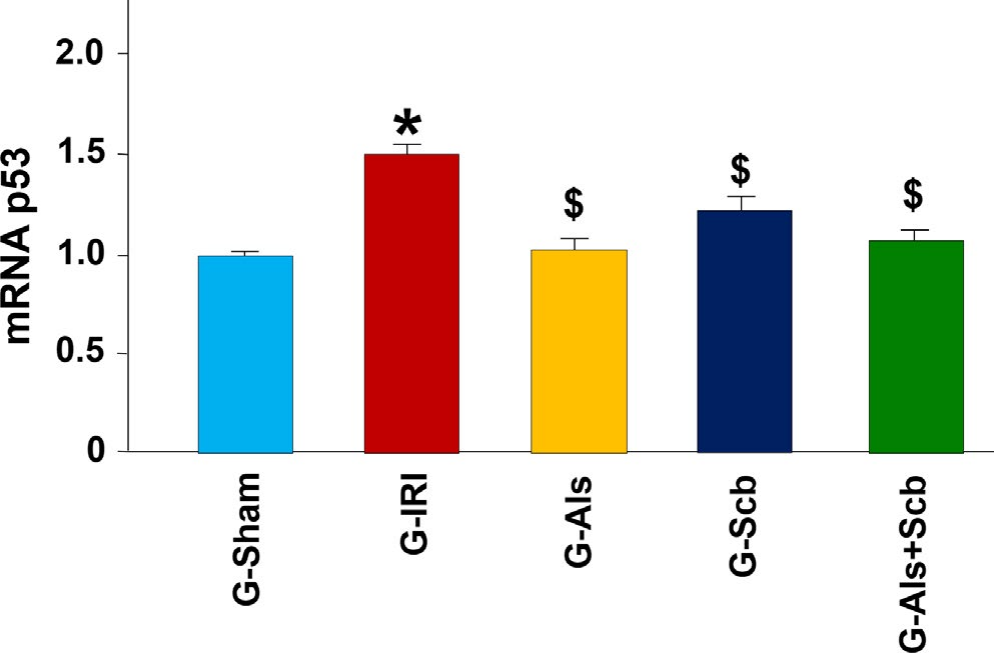
The gene expression of the pro‐apoptotic p53 gene in all groups. Values represent mean ± SEM. _*_ and $ indicate statistical significance when compared to the G‐Sham and G‐IRI, respectively

Similar findings were obtained in pro‐inflammatory, pro‐fibrotic, and pro‐apoptotic cytokines as shown in Figures 3 and 4. For example, IRI caused an increase in the gene expression of TGF‐β1 (1.98 ± 0.21 vs. 1.00 ± 0.06, *p* = 0.001). Aliskiren, sacubitril, and the combination of aliskiren and sacubitril significantly attenuated this increase in expression (1.01 ± 0.09 (*p* = 0.005), 1.07 ± 0.05 (*p* = 0.006), and 1.05 ± 0.13 (*p* = 0.006), respectively). Similar findings were obtained in the gene expression of PAI‐1, MCP‐1, and p53 (Figures 3 and 4). In all these markers, the gene expression was not significantly different when the combination therapy was compared to each agent alone (*p* > 0.05 for all comparisons).

### 3.2 Histological studies

**FIGURE 5:**
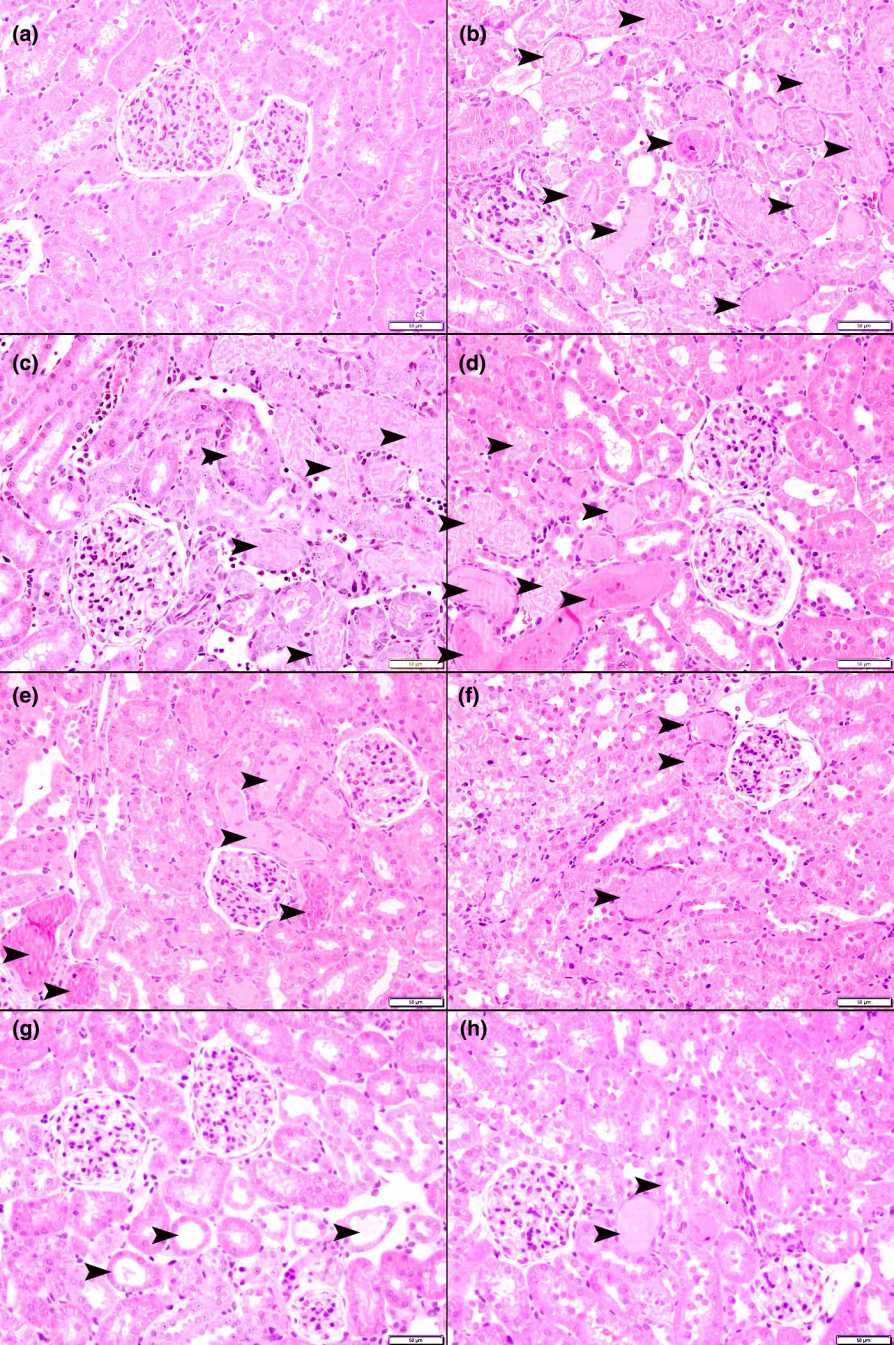
The histological features in all the experimental groups. (a) The kidneys in G‐Sham showing normal architecture and histology. (b) represents the histological features in the G‐IRI with acute tubular necrosis (arrowhead) with dilated tubules and intratubular secretion. (c&d, e&f, and g&h) show the histological features in G‐Scb, G‐Als, and G‐Als+Scb, respectively with variable degrees of acute tubular necrosis. Among the three groups, the least degree of acute tubular necrosis, is shown in G‐Als + Scb

**FIGURE 6:**
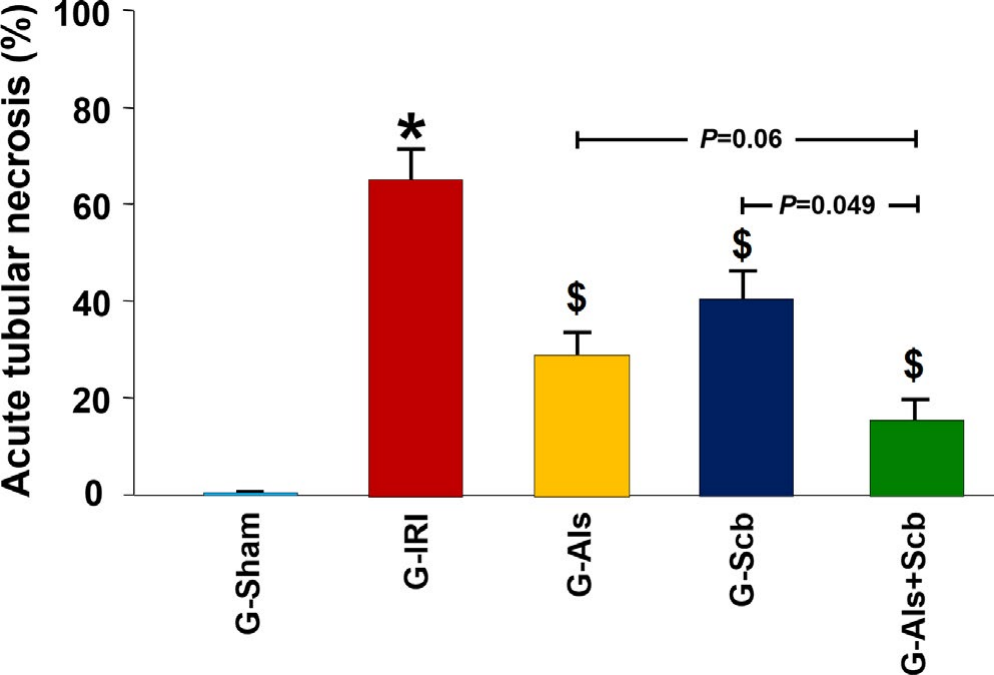
The degree of acute tubular necrosis (in percentage) in all the groups. _*_ and $ indicate statistical significance when compared to the G‐Sham and G‐IRI, respectively

As shown in Figure 5a, the kidneys in G‐Sham had normal architecture and histology (score 0). IRI caused acute tubular necrosis which was occupying 63 ± 7% of the fields (score 3; Figure 5b). This was associated with tubular dilation and intra‐luminal necrotic material, loss of proximal tubules brush border, mixed inflammatory cells infiltration of the interstitium, and focal interstitial edema. In both G‐Scb and G‐Als, there were significant reductions in the extent of acute tubular necrosis. It occupied 41 ± 6% of the fields in G‐Scb (*p* = 0.05 when compared to G‐IRI; Figure 5c,d) and 28 ± 4% of the field in G‐Als (*p* = 0.005 vs. G‐IRI; Figure 5e,f). (Score 2 for both). The combination of aliskiren and sacubitril reduced the degree of acute tubular necrosis to 15 ± 5% of the filed (score=1; (*p* = 0.0005 vs. G‐IRI; Figure 5g,h). The extent of acute tubular necrosis in G‐Als+Scb was lower than the extent in G‐Scb and G‐Als (Figure 6).

## 4 DISCUSSION

In this study, we have investigated for the first time, the effect of neprilysin inhibition and the effect of inhibiting both neprilysin and renin inhibitions on the renal dysfunction following IRI. We have shown that neprilysin inhibition by sacubitril or renin inhibition by aliskiren had independently attenuated the IRI‐induced alterations in the serum creatinine, creatinine clearance, urinary albumin leakage, the gene expression of markers of acute renal injury, pro‐inflammatory, pro‐fibrotic, and pro‐apoptotic cytokines and histological features. We have also demonstrated that the combination of these two agents had stronger protective effects compared to each one alone.

The superior effect of the combination of RAS blockade and neprilysin inhibition has been previously shown in other conditions (Damman et al., 2018; Habibi et al., 2019; Hubers & Brown, 2016; Packer et al., 2018). For instance, in an animal model of subtotal nephrectomy‐induced chronic kidney disease, a superior reno‐protective effect was observed with the combination of sacubitril and valsartan compared to valsartan monotherapy (Jing et al., 2017; Ushijima et al., 2017). In a more recent study using the same combination in an obesity model in the rat, Habibi el al have also demonstrated a superior effect of the combination therapy (Habibi et al., 2019) in attenuating the alterations in some parameters such as the podocyte and tubular mitochondrial ultrastructure, although the combination was not superior to valsartan monotherapy in some other parameters. The findings in Habibi el al study were similar, in principle, to the findings in this study. The combination of aliskiren and sacubitril was more effective than each drug in attenuating the degree of acute tubular necrosis as well as the alterations in the serum creatinine and creatinine clearance.

The lack of superiority of the combination therapy over both monotherapies in attenuating the alteration in some of the parameters such as the gene expression of the pro‐inflammatory markers was unlikely due to the lower effectiveness of aliskiren compared to other inhibitors such as valsartan which was used in the previous studies (Habibi et al., 2019; Jing et al., 2017; Ushijima et al., 2017). In this regard, aliskiren was shown to be as effective as other angiotensin receptor blockers especially in hypertension and diabetic nephropathy (Gao et al., 2011; Persson et al., 2009). One possible reason for the superiority of the combination therapy is the use of reasonably good dose of sacubitril and hence this could have led to increased production of the vasoconstrictive endothelin‐1 (*vide infra*) and hence counterbalancing the benefit of adding sacubitril to aliskiren (Roksnoer et al., 2018). Other possibilities include the difference in the nature of the alterations in different pathological conditions, different species or even the difference in the time of measuring certain parameters or mediators following the renal insult. Regardless, this research and the previous reports indicate that, whether the RAS is blocked at the first limiting step, that is, by renin inhibition, by angiotensin‐converting enzyme inhibitors or by angiotensin receptor blockers, the combination of RAS and neprilysin inhibition is more effective than either inhibition alone.

In this study, the slightly milder protective effect of neprilysin inhibition when compared to renin inhibition is probably due to the fact that neprilysin did not only mediate the degradation of natriuretic peptides (mainly ANP) but it also degraded other vaso‐constrictive compounds such as angiotensin II and Endothelin‐1 (Ando et al., 1995; Ferro et al., 1998), and hence dampening the effect of neprilysin inhibition. The other possibility for this milder effect might be due to the effect of neprilysin in the alternative RAS vasodilator pathway. In this regard, neprilysin has been shown to be a major agent in the production of Ang‐(1–7) which counterbalances the biological effects of the classical RAS‐axis (Domenig et al., 2016).

It is not known from this study if a different dose of sacubitril would have also caused the same or more degree of attenuation in the IRI‐induced renal dysfunction. In this respect, there is an evidence to suggest that different degrees of neprilysin inhibition might result in different actions. For instance, in a hypertensive rat model, Roksnoer and colleagues have shown that small doses of the neprilysin inhibitor thiorphan was more cardio‐protective compared to higher doses (Roksnoer et al., 2018). This was thought to be due to the generation of more vaso‐constrictive endothelin‐1 with higher doses. Whether the same applies to sacubitril and the IRI is difficult to ascertain from the current data and requires more studies.

Although the exact mechanism for the protective effects observed using aliskiren, sacubitril or both cannot be elucidated from this study, it appears that both agents resulted in an attenuation in the inflammatory process as demonstrated by the significant attenuation in the pro‐inflammatory and pro‐fibrotic cytokines including TGF‐β1. However, more studies are required to clarify this issue further.

In conclusion, RAS blockade by the use of the renin inhibitor aliskiren or neprilysin inhibition by sacubitril separately led to significant attenuation in the renal IRI‐induced renal dysfunction. The combination of aliskiren and sacubitril in IRI was more effective than either one alone.

## CONFLICT OF INTEREST

None of the authors has any conflict of interest to disclose.

## AUTHORS’ CONTRIBUTIONS

Fayez T. Hammad involved in project development, data collection and/or management, data analysis, manuscript writing/editing, and approval of the final version of the manuscript. Suhail Al‐Salam involved in data collection and/or management, data analysis, manuscript writing/editing, approval of the final version of the manuscript. Sarah S. AlZaabi, Awwab F. Hammad, Javed Yasin, and Maryam M. Alfalasi involved in data collection and/or management, manuscript editing, and approval of the final version of the manuscript. Loay Lubbad involved in development, data collection and/or management, manuscript editing, approval of the final version of the manuscript.
